# A Pilot Randomized Trial to Compare Polyuria and Polydipsia during a Short Course of Prednisolone or Methylprednisolone in Dogs with Atopic Dermatitis

**DOI:** 10.3390/vetsci9090490

**Published:** 2022-09-09

**Authors:** Viktorija Lokianskiene, Kerstin Bergvall, Thierry Olivry

**Affiliations:** 1 VetPet LT, Sodu g. 19, 55167 Jonava, Lithuania; 2 Faculty of Veterinary Medicine and Animal Husbandry, Swedish University of Agricultural Sciences, P.O. Box 7054, 75007 Uppsala, Sweden; 3 Department of Clinical Sciences, College of Veterinary Medicine, NC State University, 1060 William Moore Drive, Raleigh, NC 27607, USA

**Keywords:** atopic dermatitis, dog, methylprednisolone, prednisolone, polyuria–polydipsia

## Abstract

**Simple Summary:**

Glucocorticoids (a.k.a. steroids) are often used to treat allergic skin diseases in dogs, but they commonly cause side-effects, such as increased drinking (polydipsia) and urination (polyuria). Veterinarians have long believed that the steroid methylprednisolone causes less drinking and urination than prednisolone. We performed a clinical trial in which dogs with atopic dermatitis were treated either with prednisolone or methylprednisolone at the beginning of an elimination diet. After two weeks, the owners did not notice any significant increase in drinking. Most dogs had a reduction in the specific gravity (that is the density) of the urine, which signals a more diluted and abundant urine, but there was no significant difference between the two steroids for urine dilution. In conclusion, there were no significant changes in either drinking and urine dilution when giving prednisolone or methylprednisolone to allergic dogs for two weeks. A longer treatment duration or higher doses might give different results, however.

**Abstract:**

Glucocorticoids are widely used to treat canine allergic disorders, but they frequently cause polyuria and polydipsia (PUPD). At equipotent dosages, oral methylprednisolone is believed to cause less PUPD than prednisolone. We performed a pilot randomized, open, parallel trial with 22 dogs with nonseasonal AD receiving either prednisolone or methylprednisolone at equipotent dosages, once daily for 14 days during the first phase of a restriction–provocation dietary trial. Before and on days 3, 7, and 14 after starting the glucocorticoids, owners estimated water consumption for 24 h. On the same days and before the glucocorticoid was given, owners collected the first-morning urine to determine the urine specific gravity (USG). There were no significant differences between the prednisolone and methylprednisolone groups on days 3, 7, and 14 when comparing the changes in water intake from baseline. Most dogs from both groups exhibited a slight reduction in USG during the study. Still, there was no significant difference in USG changes between the groups on any of these three reevaluation days. In conclusion, the administration of two weeks of oral prednisolone and methylprednisolone at equipotent anti-inflammatory dosages at the beginning of an elimination diet did not lead to significant differences in water intake and USG.

## 1. Introduction

In dogs, cats, horses, and humans, the treatment of acute flares of allergic skin diseases often calls for the prescription of oral glucocorticoids, such as prednisolone. Despite their rapid antiallergic and antipruritic effects, the administration of oral glucocorticoids results in common side effects, with polyuria and polydipsia (PUPD) being most frequently seen [1]. Such unpleasant side effects often result in poor owner adherence to the prescribed treatment’s dose or duration.

Methylprednisolone and prednisolone are synthetic glucocorticoids that are common first-choice drugs to treat allergic, inflammatory, or pruritic skin diseases. These two glucocorticoids are related, with methylprednisolone being slightly more potent than prednisolone, resulting in a lower dosage of the former (4 mg) being considered equipotent to 5 mg of the latter [2]. These two compounds exhibit both glucocorticoid and mineralocorticoid properties with methylprednisolone having less mineralocorticoid activity [3].

For decades, veterinarians have commonly prescribed methylprednisolone rather than prednisolone to help reduce the risk of clinical PUPD. Unfortunately, we could not find any published evidence to support the dogma that using methylprednisolone rather than prednisolone would decrease this adverse event.

Our objective was to test the hypothesis that, when compared to oral prednisolone, an equivalent dose of methylprednisolone causes a smaller increase in water consumption and urine production in dogs with allergic dermatitis.

This trial was reported following the recommendations of the CONSORT statement (http://www.consort-statement.org, page last accessed on 7 September 2022).

## 2. Materials and Methods

### 2.1. Ethics

Owners gave verbal consent to participate in this trial, which uses best clinical practice standards to treat canine AD [4]. Because the short-term administration of oral glucocorticoids during a flare of AD and the initiation of a restrictive dietary trial are standard of care for this disease, approval by the regulatory agency in Lithuania was unnecessary.

### 2.2. Trial Design

This study was a pilot, randomized, open, 1:1 parallel trial lasting 14 days, with two groups of dogs.

### 2.3. Participants

We enrolled client-owned dogs between 1 and 6 years of age, deemed healthy, except for clinical signs consistent with nonseasonal AD. To reduce any possible seasonal variability in water intake, all dogs were enrolled after summer’s end.

Dogs were selected when entering the restrictive phase of a dietary trial to rule out the diagnosis of food-induced AD. Dogs should not have been on any medication known to induce polyuria or polydipsia (glucocorticoids, diuretics, anticonvulsants (e.g., phenobarbital), levothyroxine, and salt supplementation), or fluid therapy for one month preceding and throughout the study. Finally, there should not be more than one pet in the household with access to the water bowl, which was refilled as needed during the day. Pregnant or lactating females and dogs with concurrent systemic diseases were also excluded.

### 2.4. Interventions

As an elimination diet, we chose to give dogs the same commercial pet food (Anallergenic, Royal Canin, Aimargues, France), thus preventing any variation in dietary factors (e.g., salt) that could affect water intake; they did not receive any other foods, treats, antiallergic drugs, or topical glucocorticoids during this short trial.

Following the recent publication recommending the use of a short course of oral glucocorticoids when starting an elimination diet [5], dogs were randomized to receive either prednisolone (Prednisolon-Richter, Gedeon Richter, Budapest, Hungary—Group P) at 0.5 ± 0.1 mg/kg or with methylprednisolone (Medrol, Pfizer, Ascoli Piceno, Italy—Group MP) at 0.4 ± 0.1 mg/kg; both drugs were given once daily for 14 days after the morning meal.

### 2.5. Outcomes

Client-owned dogs had a thorough history, physical, and dermatological exami-nation performed by the same primary investigator upon enrollment during Visit 1 (V1).

Owners were given a form to record treatment and side effects. They were shown how to estimate 24 h water consumption and record it before starting the glucocorticoids, and on days 3, 7, and 14 (±1) after that.

Owners were also shown how to collect the first-morning urine, which was performed on the same days as above. Immediately after collection, urine samples were placed in a sterile urine container and stored in the refrigerator until analyzed. After two to three weeks, dogs returned to the clinic (V2) for re-examination by the same clinician. During that visit, owners delivered the completed forms and all four urine containers to the investigator. The stability of USG after the three-week refrigeration of urine samples had been verified beforehand by a pilot study of more than 10 dogs (our unpublished observations).

As a substitute for urine volume calculation, we chose to determine the USG using a traditional handheld analog refractometer (JAK Marketing Limited, Sheriff Hutton, York, England) [6]. Before every measurement, we calibrated the refractometer to ensure that a reading of 1.000 was obtained with distilled water warmed up to room temperature before testing. As the presence of large amounts of protein and glucose can alter the USG [7], we first performed a urinary dipstick test to ensure that there was no proteinuria or glucosuria at the time of inclusion.

### 2.6. Sample Size Estimation

Using a free online calculator, we determined the sample size (http://powerandsamplesize.com; page last accessed on 7 September 2022). We assumed that dogs treated with prednisolone would be polydipsic with a mean daily water intake of 125 mL/kg with a standard deviation of 20%. Following our hypothesis that methylprednisolone-receiving dogs would have a lower risk of polydipsia, we estimated their mean water intake to be at most 100 mL/kg with a standard deviation of 20%. Using these values, we determined that we needed to enter at least 11 dogs per group for this study to have an 80% power to detect a significant difference in water intake between groups at *p* = 0.05.

### 2.7. Randomization

Dogs were randomly allocated 1:1 into the P or MP groups using an online coin-flip generator (www.random.org; page last accessed on 7 September 2022). To ensure a similar range of weights in both treatment groups, we used blocks of two dogs (one in each group) within four ranges of weight: 0 to 10 kg (small dogs), 11 to 25 kg (medium), 26 to 39 kg (large), and 40 kg and heavier (giant). When a dog was randomized to one treatment based on a weight category, the next dog within the same weight range was automatically given the other intervention to complete that block. The owners, who measured the water intake, were not blinded to the identity of the glucocorticoid given, but they were not explicitly told that any difference was expected between two treatments.

### 2.8. Statistical Methods

We compared the percentage change from baseline values between groups on each re-evaluation visit using two-tailed Mann–Whitney tests. We used GraphPad’s Prism 7 for all these calculations (Graphpad, San Diego, CA, USA).

## 3. Results

### 3.1. Recruitment and Numbers Analyzed

Between the end of August 2021 and that of January 2022, we enrolled 30 dogs with nonseasonal signs of AD, but later excluded 9 dogs for reasons specified in Figure S1. Of the remaining 21 dogs, 10 had received prednisolone and 11 methylprednisolone when starting their elimination diet for evaluating food-induced AD.

### 3.2. Baseline Data

There were no significant differences in ages (*p* = 0.243), weights (*p* = 0.932), or sex distribution between the two groups (*p* = 0.198; Table S1). The categories of dogs, based on their weight, were similar between the two groups (Figure S1).

### 3.3. Outcomes

**Figure 1:**
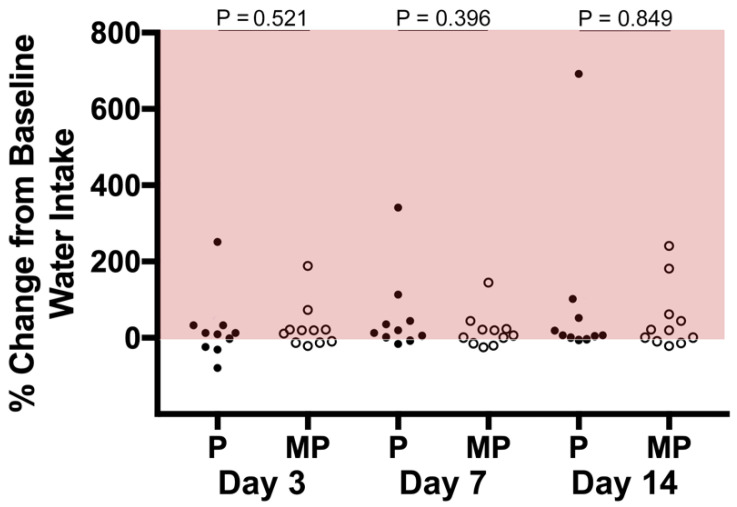
Percentage change from baseline water intake. The pink area indicates no change or increases in water consumption.

None of the dogs from either group exhibited polydipsia (above 100 mL/kg/day) during the study. In Figure 1, we represent the changes in water consumption from baseline. In the 10 dogs from the P group, increases in water intake were seen in 6 (60%), 8 (80%), and 8 dogs (80%) on days 3, 7, and 14, respectively. In the 11 dogs from the MP group, such increases compared to baseline were observed in 7 (64%), 6 (55%), and 6 dogs (55%) at the same time points. When comparing the changes in water intake from baseline, there were no significant differences between the P and MP groups on days 3, 7, or 14 (Mann–Whitney tests; *p* = 0.521, 0.396, and 0.849, respectively).

**Figure 2:**
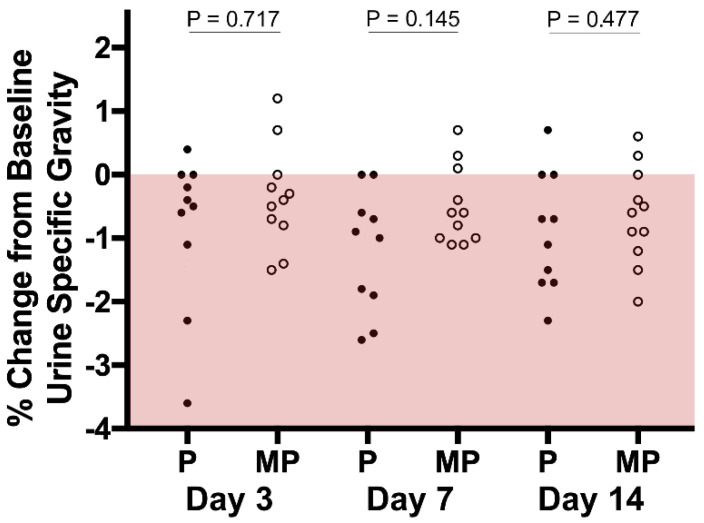
Percentage change from baseline urine specific gravity. The pink area indicates no change or decreases in USG.

In this study, we considered a normal USG as being higher than 1.015 (http://www.iris-kidney.com/education/urine_specific_gravity.html; page last accessed on 7 September 2022). All dogs had normally concentrated urine at baseline. Most dogs from both groups exhibited a slight reduction in USG during the study (Figure 2). Nevertheless, there was no significant difference in the change in USG between the P and MP groups on either day 3 (*p* = 0.717), day 7 (*p* = 0.145), or day 14 (*p* = 0.477).

### 3.4. Adverse Events

In dogs that finished this study, adverse events were not reported by the owner, except for one dog treated with prednisolone that exhibited polyphagia (Dog P7).

## 4. Discussion

To the best of the author’s knowledge, this is the first trial comparing changes in water consumption and USG after treating dogs with two related glucocorticoids.

In our two-week pilot study, prednisolone and methylprednisolone led to minor increases in water intake in about half of the dogs. Still, there were no significant differences between groups at any of the three evaluation points, thus suggesting that the short-term treatment with anti-inflammatory dosages of methylprednisolone does not reduce water consumption compared to prednisolone, at least during the first two weeks of administration.

In this trial, as we could not reliably document the urine output in client-owned dogs, we chose USGs as a surrogate biomarker expected to be inversely correlated with the urine volume. When comparing the changes from baseline USG between treatment groups, there were no significant differences at any of the urine collection times. As for water consumption, this absence of a significant difference between treatment groups suggests that methylprednisolone does not cause less polyuria than prednisolone, at least in the short term. However, we do not rule out that a longer treatment, a higher dosage, larger and heavier dogs, or a more frequent administration of the drugs would lead to more noticeable differences between these two oral glucocorticoids. The influence of these parameters should be explored in further studies.

There are two main limitations to this study. The first is the small number of dogs followed. We calculated the number of subjects enrolled in each group based on an expected increase in 25% of water intake in prednisolone-treated dogs compared to those receiving methylprednisolone. At the end of our two-week trial, however, the median water intake was only 10% higher in dogs treated with the former compared to the latter. We suspect that the lack of polydipsia development was due to the relatively low dosage and short duration of this trial. After the fact, we can assume that the number of dogs enrolled herein was likely insufficient to detect this small difference between treatments as significant.

A second limitation is that we did not monitor the urinary osmolality, which is considered the gold standard for estimating urine concentration. Nevertheless, the USG provides a fair estimate of the osmolality if the urine does not contain high amounts of protein, glucose, or other large molecules, such as radiocontrast media [7]. For this reason, we used urine dipsticks beforehand to ensure the lack of glucosuria or proteinuria. Moreover, a recent study had shown a good correlation between the USG and urine osmolality in one hundred dogs [8].

## 5. Conclusions

In this trial, the administration of two weeks of oral prednisolone and methylprednisolone at equipotent anti-inflammatory dosages at the beginning of an elimination diet did not lead to significant differences in water intake and USG used a surrogate for urine output in young adult dogs. It is unclear if these results can be extrapolated to all other (especially older) dog populations. Moreover, different glucocorticoid treatment regimens might lead to other observations and the opposite interpretation. The conclusions of our study should be verified in additional trials using the present results for power calculation and study subject number determination. In future studies, the measurement of the urine osmolality should be preferred to that of the USG for a more precise assessment of the urine concentration.

## Data Availability

Not applicable.

## Supplementary Materials

The following supporting information can be downloaded at: https://www.mdpi.com/article/10.3390/vetsci9090490/s1. Figure S1: CONSORT flow diagram; Table S1: signalment and weights of the dogs at baseline.

## References

[1] Elkholly D.A., Brodbelt D.C., Church D.B., Pelligand L., Mwacalimba K., Wright A.K., O'Neill D.G. (2020). Side effects to systemic glucocorticoid therapy in dogs under primary veterinary care in the UK. Front. Vet. Sci..

[2] Miller W.H., Griffin C.E., Campbell K. (2012). 3. Dermatologic therapy. Muller & Kirk’s Small Animal Dermatology.

[3] Liu D., Ahmet A., Ward L., Krishnamoorthy P., Mandelcorn E.D., Leigh R., Brown J.P., Cohen A., Kim H. (2013). A practical guide to the monitoring and management of the complications of systemic corticosteroid therapy. Allergy Asthma Clin. Immunol..

[4] Olivry T., DeBoer D.J., Favrot C., Jackson H.A., Mueller R.S., Nuttall T., Prélaud P. (2015). Treatment of canine atopic dermatitis: 2015 updated guidelines from the International Committee on Allergic Diseases of Animals (ICADA). BMC Vet. Res..

[5] Favrot C., Bizikova P., Fischer N., Rostaher A., Olivry T. (2019). The usefulness of short-course prednisolone during the initial phase of an elimination diet trial in dogs with food-induced atopic dermatitis. Vet. Dermatol..

[6] Rudinsky A.J., Wellman M., Tracy G., Stoltenberg L., DiBartola S.P., Chew D.J. (2019). Variability among four refractometers for the measurement of urine specific gravity and comparison with urine osmolality in dogs. Vet. Clin. Pathol..

[7] Flasar C. (2008). What is urine specific gravity. Nursing.

[8] Dossin O., Germain C., Braun J.P. (2003). Comparison of the techniques of evaluation of urine dilution/concentration in the dog. J. Vet. Med. Physiol. Pathol. Clin. Med..

