# Supplementary material for: A Pilot Randomized Trial to Compare Polyuria and Polydipsia during a Short Course of Prednisolone or Methylprednisolone in Dogs with Atopic Dermatitis
Source: Vet Sci. 2022 Sep 9;9(9):490. doi: 10.3390/vetsci9090490 (PMC9506298; doi:10.3390/vetsci9090490)
Supplement: Supplementary file 1 [file vetsci-09-00490-s001.zip › vetsci-1896214-supplementary.pdf]

Supplementary Material

Figure S1: CONSORT Flow Diagram

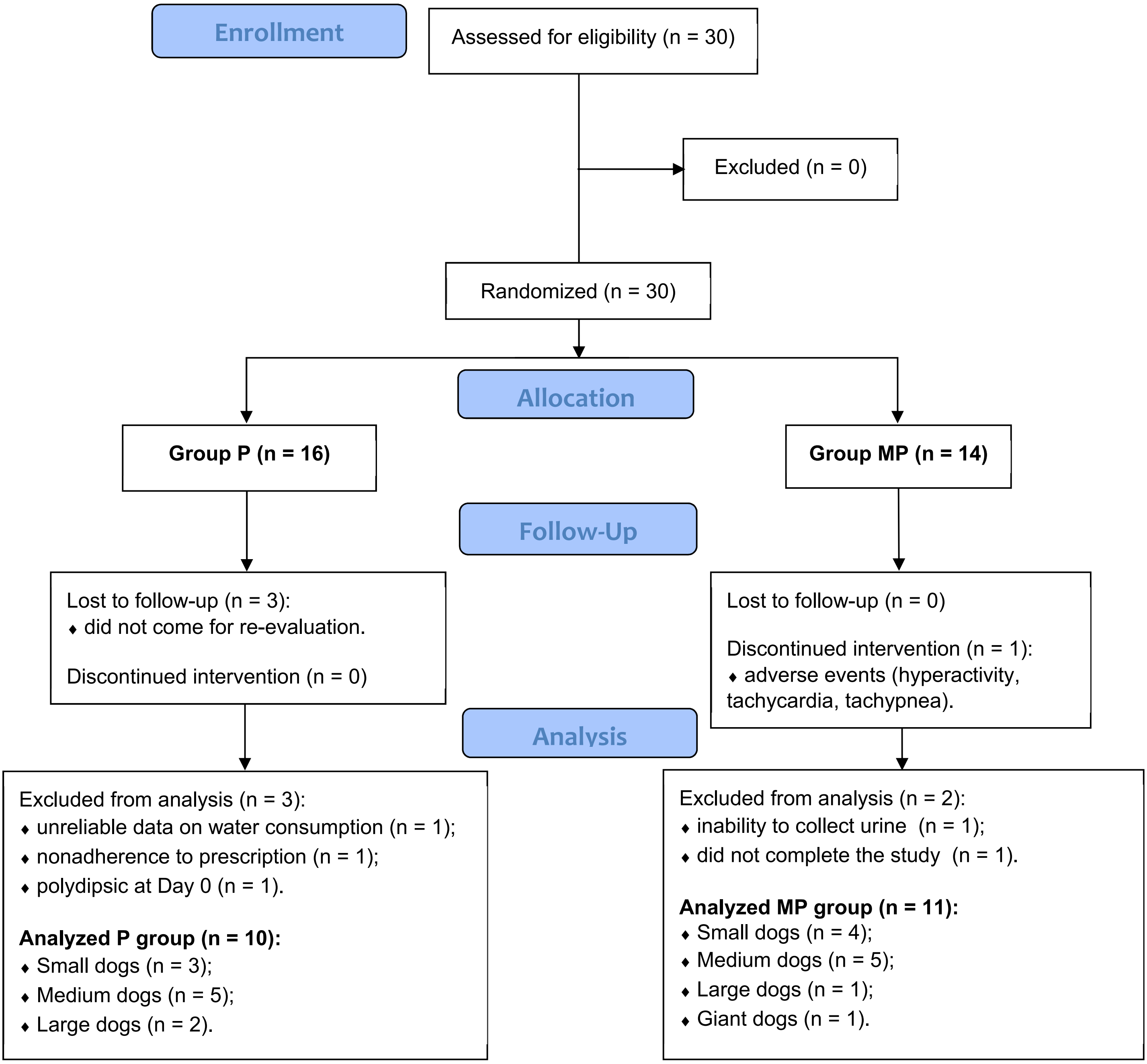

Table S1: Patient Characteristics at Baseline

Prednisolone (P) group: signalment and weights of the dogs at baseline

Dog	Breed	Age (Y)	Weight (Kg)	Sex
P1	crossbred	2.7	9.9	FI
P2	crossbred	2.2	16.6	FI
P3	crossbred	5.3	2.5	MC
P4	cockapoo	2	9.3	FS
P5	French bulldog	4.6	14.3	MI
P6	Akita inu	1.2	33.4	MI
P7	dachshund	6.5	11.5	MI
P8	crossbred	2	16.1	MI
P9	giant schnauzer	2.8	37.5	FI
P10	French bulldog	3.5	14.5	MI
	Minimum	1.2	2.5	
	Maximum	6.5	37.5	
	Median	2.8	14.4	
	Mean	3.3	16.6	

Methylprednisolone (MP) group

Dog	Breed	Age (Y)	Weight (Kg)	Sex
MP1	miniature schnauzer	2.1	6.8	FI
MP2	crossbred	2.8	9	FS
MP3	French bulldog	2.7	12.7	FI
MP4	Akita inu	1.6	22.9	FI
MP5	crossbred	2.9	19.6	FS
MP6	doberman pinscher	3.9	46.8	MI
MP7	American cocker spaniel	1.1	8.6	FI
MP8	Chinese shar Pei	3.9	22	FI
MP9	French bulldog	1.8	9.5	MI
MP10	golden retriever	1.1	39	MI
MP11	crossbred	2.1	11.5	FS
	Minimum	1.1	6.8	
	Maximum	3.9	46.8	
	Median	2.1	12.7	
	Mean	2.4	18.9	

Abbreviations: FI: female, intact; FS: female, spayed; MC: male, castrated; MI: male, intact; MP: methylprednisolone; P: prednisolone
